# A. Cornelii Celsi De Medicina Libri Octo; Ex Recensione Leonardi Targæ

**Published:** 1833

**Authors:** 


Art. VI.—*A. Cornelii Celsi de Medicina Libri octo ex recensione Leonardi Targæ.*

*The eight books of Aulus Cornelius Celsus on Medicine; from the text of Leonardus Targa.*

The excessive admiration of the ancients which, a century ago, was as general among professional men, as among the votaries of literature, has of late, so far as our profession is concerned, very much subsided. A familiar acquaintance with Hippocrates and Galen, may perhaps, at present, add something to the dignity of the professor, or the reputation of the writer of grave professional systems; but the mere practitioner derives very little importance in society from antiquated professional reading. Indeed, a prejudice seems rather to be excited against him—a kind of *blue stocking* character is assigned him that is calculated to do any thing rather than promote his interest.

We should not be disposed to blame the decision of the public in this respect, if it were founded upon a due estimate of the value of these writings. Indeed it requires one but to look into the writings of these authors to be convinced that their whole merit consists in the accurate records of disease which they have transmitted to us. They were accurate observers of symptoms; and here, as they were liable to be misled by no theory, their descriptions are true to nature. Observant also of the influence of external agents upon the general health, their works contain many valuable precepts respecting diet and regimen, but as physicians it is impossible to conceive of the crudeness of their opinions, and the absurdity of their practice. Yet, to a mind imbued with a proper degree of love for his profession, the opinions of those who have been distinguished before us in the same field of investigation, ought not be destitute of interest.

The practical instruction they convey does not, it is true, entitle them to occupy a place among the graver studies of the severe student; but none certainly can regret having occasionally devoted an hour of relaxation to the writings of men who have occupied so large a place in our profession. This is the position we would assign them. As a liberal relaxation from the studies or labors of the profession—as a means of enabling us to estimate more accurately, and to retain more firmly the modern improvements of our profession, by tracing the progress of improvement from the infancy of the art, they have a value that may be differently estimated, but must be acknowledged by all.

Entertaining this view of their importance, we of course differ altogether from those who would make them a part of the elementary studies of the young physician. The course said to have been pursued by Dr. Rush, of commencing with Hippocrates, and studying, successively, the different authors who have been distinguished since his day, is deserving of any thing rather than imitation. From such a course of study we would expect nothing but confused, incoherent and incorrect ideas. To the mind of the student, science should be presented in its simplest and most accurate form, divested of every thing calculated to divert the attention from established principles, or to encourage the imagination at the expense of the judgment. When, from his familiarity with elementary principles, the student is capable of distinguishing the valuable from the useless, and of placing a proper estimate upon the visionary speculations with which the records of medicine abound, then may he be permitted to extend his reading to a greater variety of authors. Even this, however, should be done with the greatest caution, or, if he do not form habits of speculation that will tend greatly to obstruct his progress in knowledge, by prepossessing his mind with false and visionary notions, and by directing it to false objects of pursuit, he will at least have reason to regret that much valuable time has been spent in the acquisition of what will prove perfectly useless. These remarks apply to a certain extent to authors of a much later date than those of which we are now speaking. Every physician will recollect how fascinated he once was with the speculations of Cullen,—speculations which he must now acknowledge do not even serve for empty pretences to amuse the vulgar by a show of knowledge. It is time, however, for us to return from this digression to the consideration of the volume before us.

Celsus is supposed to have flourished during the reign of Augustus. Whether he was ever engaged in the practice of medicine, or whether he prosecuted the study for his private amusement, is a disputed question, and, since the practice of medicine at the time in which he wrote, was so very imperfect that it is impossible to form an opinion from the internal evidence of the work, the subject must remain involved in obscurity.

However this may be, his work has justly been considered as one of the most valuable of the medical treatises which has descended to us from antiquity. The perspicuity, conciseness, and classical Latinity of his style prove him to have been as accomplished in the literature, as he was familiar with the medical knowledge of the age in which he lived.

Celsus informs, that in the origin of the healing art, its exercise was confined to the practice of surgery; disease being referred to the anger of the gods, its removal was sought for in the various modes of appeasing them which superstition pointed out. In later times as luxury and refinement introduced a greater variety of diseases, the art of healing was connected with the study of philosophy. To Hippocrates is ascribed the honor of having been the first to elevate it to the rank of a distinct profession.

The professors of medicine were, at a very early period of its history, divided into two great classes—those who believed that experience was the only rational foundation of practice, and those who believed, that having first ascertained, as far as possible the laws of animal life in the healthy condition of the body, nothing remained but to exercise their reason in applying the knowledge thus acquired to the cure of the diseases.

The latter class were great advocates for the study of anatomy, thinking, very justly according to our notions, that a man would be better qualified for curing the disease of an organ, the structure and physiology of which he knew something about. Some of this sect were not content with the knowledge they acquired by the examination of dead bodies, but Herophilus and Erasistratus opened the bodies of the criminals while yet alive, in order that they might observe "the color, form, magnitude, arrangement, texture, and feel" of the organs; thinking it impossible that a physician could pretend to cure the disease of a part of which he was entirely ignorant. Nor did they consider these investigations cruel, since it was perfectly reasonable, that, "by the punishment of a few criminals, means of relief should be sought for innocent persons throughout all time." An admirable comment upon the vivisectors of our own days!

To this the Empirics replied that the search after the hidden and obscure causes of vital action was fruitless, because nature was incomprehensible; that the discord which prevailed among those who professed to teach wisdom to others, rendered it certain that reason could never establish successful principles of practice, and therefore credit ought to be yielded to the authority of no one until it was established by the successful treatment of disease; that a knowledge of the nature of disease, as in the case of wounds where the cause was apparent, did not naturally suggest the appropriate remedy, still less could it do this in the case of the internal organs, where the cause of disease could be, at best, but matter of conjecture; and that since the whole subject was thus obscure and incomprehensible, our dependence must be placed in the sure result of experience; in medicine as in other arts, skill was acquired by practice and observation, and not by disputation. They further contended that the foundation of the healing art was laid in observation, and that observing men having found that certain things were useful in disease, and others injurious, directed the sick to practice the one or avoid the other, until general principles were established; and that after this, men began to discuss the reason and nature of diseases and their remedies, and thus, theory in medicine was subsequent to practice; and if the teaching of reason is in coincidence with experience, it is superfluous, if in opposition, it is false. This process of argumentation was followed up by the bold assumption that for all known diseases, remedies were already discovered, and therefore, unless new diseases appeared, no new remedies were desired. If, however, new kinds of disease should appear, they might be compared with others that were known, and thus analogy would suggest the appropriate method of treatment.

With arguments like these, obscured by appeals to prejudice, the rival sects contended bitterly against one another, each more anxious to shew the absurdity of the opinions of his antagonist, and load him with odium, than to prove himself in the right. Accustomed as we are to assign to theory and observation their appropriate sphere, and properly to estimate the due value of each, we may smile at the want of point which characterizes this contest; but it is a fair specimen of the mixture of truth and error which imparts obstinacy and bitterness to every dispute in which the passions of men become enlisted.

The two first books of this volume are devoted to the general principles of dietetics; the rules of preserving health; the difference of temperament and constitution; the influence of age, sex, and occupation; the signs of health and disease; the qualities of different kinds of food; and the virtues of different kinds of medicine.

The third and fourth books are devoted to the practice of Medicine; of which, fevers, then as now, constituted a most important part.

It would be unnecessary to follow the author through his description of the symptoms of fevers; indeed he says so little upon the subject that it is almost impossible to avoid the conclusion that he could not himself have been very conversant with them. There is one point, however, to which we refer with pleasure, because, although a very important one in the history of the autumnal fevers of the Southern States, we do not know that it has been noticed by any author except Doctor Jackson. It is this, that in the double tertian form of fever which prevails there, almost universally the milder paroxysm is followed by a restless and uncomfortable night; while, on the other hand, the more violent paroxysm which occurs on the uneven days, has a tendency to a more perfect remission, so that the night is spent in comparative comfort. The following is his language:

"It almost universally happens that the most comfortable night follows the most severe paroxysm, and thus it follows that the most distressing night precedes the severest paroxysm."

In the cure of fever, Celsus made but little use of medicine. "I think," says he, "that potions of medicine are to be given, and the bowels to be moved but seldom; and even this is not to be done in such a manner as that the strength may be wasted, for the principal danger arises from debility." His principal confidence was placed in a proper regulation of the diet; and for this purpose, rules are laid down with a great deal of minuteness. His principal anxiety was to select a time to give food during the intermission, and at as long an interval as possible before the accession of the next paroxysm. For this purpose it was withheld almost entirely for the first three or four days until the type of the fever was accurately ascertained, and then, if the interval between the paroxysms was short, it was given soon after the close of that paroxysm which was followed by the longest intermission. The food which he recommended was unirritating enough to satisfy the most rigid Broussaian. He esteemed it, however, a matter of very great importance to preserve the strength by nourishment of some kind, and conceived that this importance increased with the debility which accompanied the fever; thus it was considered necessary to commence the use of food at an earlier period, in pestilential fevers, in warm climates, in the summer season, and to very young persons.

His apprehensions respecting the use of water were very great. The more violent the fever the more dangerous was it to drink water. No drink was to be given on the first day, unless the fever should so far subside as that the patient could take nourishment; and in the mean time, he was to be comforted with the assurance that the thirst would cease when the paroxysm subsided, and that he would sooner cease to thirst if he drank nothing at all. Nor was any to be allowed on the second day, if the fever did not so far subside as to permit of the patient's taking nourishment; except under some circumstances, when the patient was oppressed with bile or *crudity*, some water might be given to dilate the irritating contents of the stomach.

It was an important consideration whether the body was *constricted* or *relaxed.* If the former, then the bowels were to be moved, the urinary organs to be excited, and perspiration to be elicited in every possible manner. In this form of fever, blood was to be drawn, the body to be agitated by the most violent kinds of passive exercise, and the system to be subdued by fasting, thirst and famine, the warm bath was to be frequently used, the food to be light and sparing, and the drink more abundant than under other circumstances. On the contrary, if the body is *relaxed*, the patient is to be indulged in quiet and repose, and the discharges are to be restrained by remedies appropriate to the organ from which they come.

The class of medicines called *Tonics* seems to have been entirely unknown to the ancients. They consequently possessed no means of cutting short the course of a fever, nor did they think it safe to do so if in their power. It might be supposed that with the limited remedial agents they had at their control, they would have entertained no apprehensions lest the disease should have been cured too speedily. This, however, was actually the case. Under the impression that a disease too speedily removed was liable to return, they reprobated the practice of a speedy cure. Indeed the whole amount of their interference appears to have been to bring the system into the most favorable condition to permit nature to bring about a solution of the disease by her own efforts. The opinion that a disease could only be safely cured by the *vis medicatrix naturæ*, and that its different stages until, nature brings about its resolution, follow each other in regular succession, is an opinion that mankind have ever been prone to entertain favorably. It is unnecessary at this time to say how completely such an opinion is refuted by the present achievements of our profession; nor is it necessary to remark how fatal such an hypothesis must be to all vigorous practice, and to every prospect of improving the resources of the art of healing.

Having disposed of the subject of fevers, it would startle one, accustomed to the cumbrous nosology of the present day, to see how small is the catalogue of diseases of which Celsus treats.

Some thirty or forty diseases, and many of these mere symptoms, compose his whole catalogue. Ignorant as they were of pathology, anatomy and physiology, they cannot be supposed to offer any think worthy of notice in their practice or their speculations. The only active remedy they employed, with the exception of purging in mania, was blood-letting. This remedy was sometimes, although with great reluctance employed in fever, but it was principally confined to cases of inflammation. They were acquainted both with cupping and bleeding, and of the different circumstances under which one or the other ought to be applied, appear to have formed a very correct idea.

Surgery, as practised at present, was divided into two departments; one treating of those forms of disease or of injury which the surgeon was called upon to relieve, the other in which he was required to use the knife.

If a minute division of external agents calculated to produce different effects upon the body was all that is necessary to produce successful surgical practice, there would have been nothing wanting to the cotemporaries of Celsus. Medicaments they had in abundance, to suppress hemorrhage, to glutinate wounds, to concoct them and promote the formation of pus, to open them, to purge them, to corrode them, to form crusts upon ulcers, to discuss tumors, to cause them to suppurate, to promote the granulation of ulcers, &c. These medicaments were variously and operosely compounded, according to the whim of the chirurgeon or upon the authority of some great name. The treatment of wounds by Celsus appears to have been judicious, and the symptoms arising from the wounds of the different viscera are very accurately laid down. He appears to have been fully aware of the utility of treating recent wounds by simple dressings, but the prejudice of the times in favor of a more complicated and artificial mode of treatment, were too great to permit it to be carried into general practice. He was acquainted with the use of the ligature for the suppression of hemorrhage, since he used it in some operations, especially in extirpation of the testicle; but in the hemorrhage arising from wounds he appears to have relied entirely upon pressure applied to the mouth of the wound.

The two last chapters are devoted to operative surgery; and with a brief account of some of their most important operations we shall close this review.

In the days of Celsus, surgery was already made a distinct branch of the profession, and practised by a body of men who professed to devote themselves to it exclusively; though, as the limits of the profession were not very extensive, it is not without reason that he praises those whose information embraced all the different branches of medicine. With many of the different operations upon the eye they were familiar. The operation of couching for cataract is well described by Celsus, who also directs that if the cataract rises again, it should be broken up and cut to pieces with the needle. With the account of the operation he gives a description of the eye, upon which we merely notice, as an instance of their careless observation, the opinion that the color of the eye was determined by that of the vitreous humor.

They removed polypus of the nose by means of an instrument shaped like a spatula, taking great care to avoid injuring the cartilages of the nose. For the cure of ozæna, he says, "some recommend that a reed should be introduced into the nostril, and through it, a heated iron wire be applied to the bone, or that the nostril should be cut up as high as the nasal bone, that the diseased part might be exposed to view and the cautery be applied more readily.

The tonsils, when permanently enlarged, were seized with a hook and excised with the scalpel, the bleeding being afterwards restrained by caustic applications. A relaxed uvula was treated in the same manner.

Umbilical hernia was treated, as it is at present, by the suture, with view to a radical cure. For scrotal or inguinal hernia, if the contents of the hernia could not be reduced by the taxis, no radical cure was proposed unless it was omental. The sac was then opened, and the protruding omentum destroyed by caustic dressings, or its circulation interrupted by the ligature. Some surgeons recommended the omentum at once to be cut away; but this practice was reprobated by Celsus as being liable to be followed by dangerous hemorrhage. All interference with strangulated hernia was considered highly dangerous, and the cure was left to nature, with such relief as venesection, fomentations, the warm bath, enemata, and low diet could afford.

Celsus is well known as the first author of antiquity whose description of the present mode of operating for the stone has reached us. The mode of operating, without sound or gorget, was rude and simple enough, and very similar to that in use among the native practitioners of India at this day. The difficulties attending the operation, and the precautions necessary are laid down with a precision that must have resulted from long practice.

In pregnant females, if the fœtus died near the period of parturition, manual assistance was supposed to be necessary for its removal. The hand of the surgeon being previously oiled, the os uteri was to be dilated by the introduction of first one finger, and then another, until the whole hand could be introduced. An attempt was then to be made, either make the head present, or to bring down the feet. If the head presented, the extraction was made with the assistance of the crotchet; if the feet, it was brought down as at the present day. The whole treatment was rash and violent in the extreme.

The treatment of piles was sufficiently bold. The indurated tumors which remain after repeated attacks, were freely removed by the knife. Bleeding piles, if small, were first included in a ligature and then laid open with a scalpel; if larger, the membrane around its base was divided before the ligature was applied. The part was then dressed with lint and cerate, and the inflammation resulting from the operation allayed by fomentations and the warm bath.

In injuries or diseases of the skull, the ancient surgeons were familiar with the use of the trephine, and appear to have anticipated some of the principles of operation which are the boast of modern surgery. For instance, respecting the immediate use of this instrument in cases of depression, Celsus says:

"In all fissures and fractures of the bone, it was customary with the older practioners to proceed at once to the use of the instruments for excision. But it is far better first to make trial of such plasters as are composed for the calvaria, and to apply any one of them alone, softened with vinegar, upon the fissured or fractured bone; then over that, to lay on a piece of linen rag somewhat wider than the wound itself, spread with the same medicine; and in addition to these, succid wool steeped in vinegar: then, to bandage the wound and open it daily, that it may be dressed in the same way for five days running: on the sixth day to foment it with a sponge and hot water, continuing the other measures. If granulation commence, and the fever have either altogether subsided, or it be but slight, if the appetite for food return and the patient sleep, we are to persevere in the use of the same local application. Afterwards, in process of time, the plaster must be softened by the addition of the cerate made from rose oil, to facilitate granulation, for used by itself it has a repressing quality. In this way, fissures are frequently filled up by a sort of callus, which serves as a cicatrix to the bone; and even more extensive fractures are agglutinated by the same callus, where they do not come in contact; and this constitutes a much better defence to the brain, than the flesh which grows over it, after partial excision of the bone. If, however, after this our first treatment, the fever be increased, the sleep short, and interrupted by tumultuous dreams, the ulcer moist, and not nourished, the glands of the neck enlarged and the pains violent, while after all these symptoms the loathing of food increases, then at last, there is a necessity for proceeding to a manuel operation with the chisel."

We question also if the following expression did not suggest to Mr. Abernethy the idea, for which he was at one time so much lauded, of scraping the bone to ascertain whether there was extravasation beneath it:

"Rarely, but yet occasionally, it happens, that the bone remains entire, while internal rupture of some one of the veins of the cerebral membrane occasions hemorrhage, and the blood coagulating excites severe pains, and in some cases blindness. Now more usually the pain is referred externally to the part which corresponds with the extravasation, and on laying open the integument there, *the bone is discovered to be pale;* therefore that too must be removed."

We shall close this article with the author's account of the operation for varicose veins of the legs—an operation, we should think, sufficiently formidable not to be very frequently repeated:

"Our next transition from these last affections is to the legs. Varices occurring in these, are easily removed. I reserved the operations required for varicose veins of the head, as well as those which form over the abdomen, that I might treat of them also in this paragraph; since they all require the same method of cure. The diseased vein then is either obliterated by cauterization, or excised by the knife. If such veins be straight, or even if situated transversely, provided they be single and of a moderate size, the cautery is preferable. If tortuous, and disposed in orbs, with a number of them interlacing each other, the better plan is to remove them. The method of cauterizing them is as follows: An incision is made through the integument by which they are covered; then, having exposed the vein, a moderate pressure is made upon it with a small blunt heated cautery, care being taken not to burn the edges of the wound, which may easily be retracted by hooks. The cauterization must be repeated throughout the whole course of the varix, at interspaces of about four digits: afterwards dressing the wound with either of the remedies proper for burns. But the incision is thus performed. The skin over the vein having been incised as before, its edges are retracted with the small hook; and the vein is completely detached on all sides from the surrounding flesh, taking care at the same time not to wound the vein itself, under that, a blunt hook is passed; and usually at the same distance as above mentioned a second under the same vein; and so on throughout the entire course of the varicose vessel, whose course is easily detected by extending the hook. This done, the vein being drawn, at one point by the hook, is divided; then, where the next hook is fixed, it is drawn up, and the excision repeated. In this way, the legs being entirely freed from the varices, the edges of the wound are approximated, and an agglutinant plaster laid over them."

For those who may not be sufficiently acquainted with the Latin to read the author in the original language, a translation has been published by Doctor Collier, of London, not very elegant nor at all times perfectly accurate, but sufficiently faithful for those who wish a general insight into the author's views.

In perusing the works of this venerable author we would caution our youthful reader against an ill-judged triumph on the superiority of modern science. Feelings of this kind are little calculated to render the perusal beneficial to him. Let him rather draw inspiration from the Genius which, with all its defects, has triumphed over the vicissitudes of nearly nineteen centuries; and that he may do this the more effectually, let him remember that all intellectual improvement is progressive, and that the era is probably not very remote when our successors will look upon the opinions of our contemporaries who may have the good fortune to outlive the present generation, with very little more respect than we now look upon those of Celsus.

